# Dynamical systems of fate and form in development

**DOI:** 10.1016/j.semcdb.2025.103620

**Published:** 2025-06-03

**Authors:** Alex M. Plum, Mattia Serra

**Affiliations:** University of California San Diego, Department of Physics, 9500 Gilman Dr, La Jolla, CA 92093, USA

**Keywords:** Dynamical systems, Morphogenesis, Pattern formation, Cell differentiation

## Abstract

Developmental biology has long drawn on dynamical systems to understand the diverging fates and the emerging form of the developing embryo. Cell differentiation and morphogenesis unfold in high-dimensional gene-expression spaces and position spaces. Yet, their stable and reproducible outcomes suggest low-dimensional geometric structures—e.g., fixed points, manifolds, and dynamic attracting and repelling structures—that organize cell trajectories in both spaces. This review surveys the history and recent advances in dynamical systems frameworks for development. We focus on techniques for extracting the organizing geometric structures of cell fate decisions and morphogenetic movements from experiments, as well as their interconnections. This unifying, dynamical systems perspective aids in rationalizing increasingly complex experimental datasets, facilitating principled dimensionality reduction and an integrated understanding of development, bridging typically distinct domains.

## 1. Introduction

Embryogenesis consists of morphogenesis and cell differentiation [1]. Each facet has a long history of incorporating mathematics, in particular geometry, as in the bifurcating chreodes of C. H. Waddington [2] and the morphological coordinate transformations of D’Arcy Thompson [3]. What began as metaphors and conceptual illustrations have recently been made mathematically rigorous in theoretical frameworks that integrate experiments. Theoretical frameworks are crucial for conceptualization, classification, and compression in biology, extracting the organizing features of life’s complex processes, enabling interpretable predictions, and bringing together scattered details [4-6]. Here, we review recent theoretical frameworks for studying the dynamics of cell differentiation and morphogenesis, with particular attention to how fate and form are integrated.

As more data becomes available, understanding development requires objective, concise descriptions of its phenomenology, onto which underlying molecular and biophysical mechanisms can be mapped [7-9]. This includes atlases of cell types [10] and their differentiation hierarchy in gene expression space [11], as well as material trajectories and cell lineages in position space. Modern experimental technologies are enabling the measurement and reconstruction of molecular profiles and morphogenetic movements across entire embryos over time [12, 13]. However, uncovering the underlying mechanisms from this data explosion, and understanding how they integrate, requires extracting the essential, organizing features of the dynamics. For cell differentiation, this means reducing high-dimensional gene-expression dynamics to simpler frameworks for cell fate decisions, where the far smaller number of cell types and transitions suggests low-dimensional fate decision dynamics. For morphogenesis, this means extracting the robust features of multicellular motion, reducing 4D velocity (3D space + time) or trajectory data into simpler kinematic structures that summarize and classify complex morphogenetic movements. Both are the purview of dynamical systems.

## 2. The embryo as a dynamical system

A dynamical system consists of a space of possible states (phase space) and rules determining where each state goes next. The progression of a system’s state over time (t) constitutes a trajectory in phase space. Advances in dynamical systems theory have provided a geometric and visual picture for understanding complicated differential equations, identifying small numbers of qualitative possibilities for the robust organization of trajectories in phase spaces given phenomenological constraints [9,14,15]. This perspective is also valuable for understanding the integrated effects of distinct mechanisms [1,16,17].

**Fig. 1.**
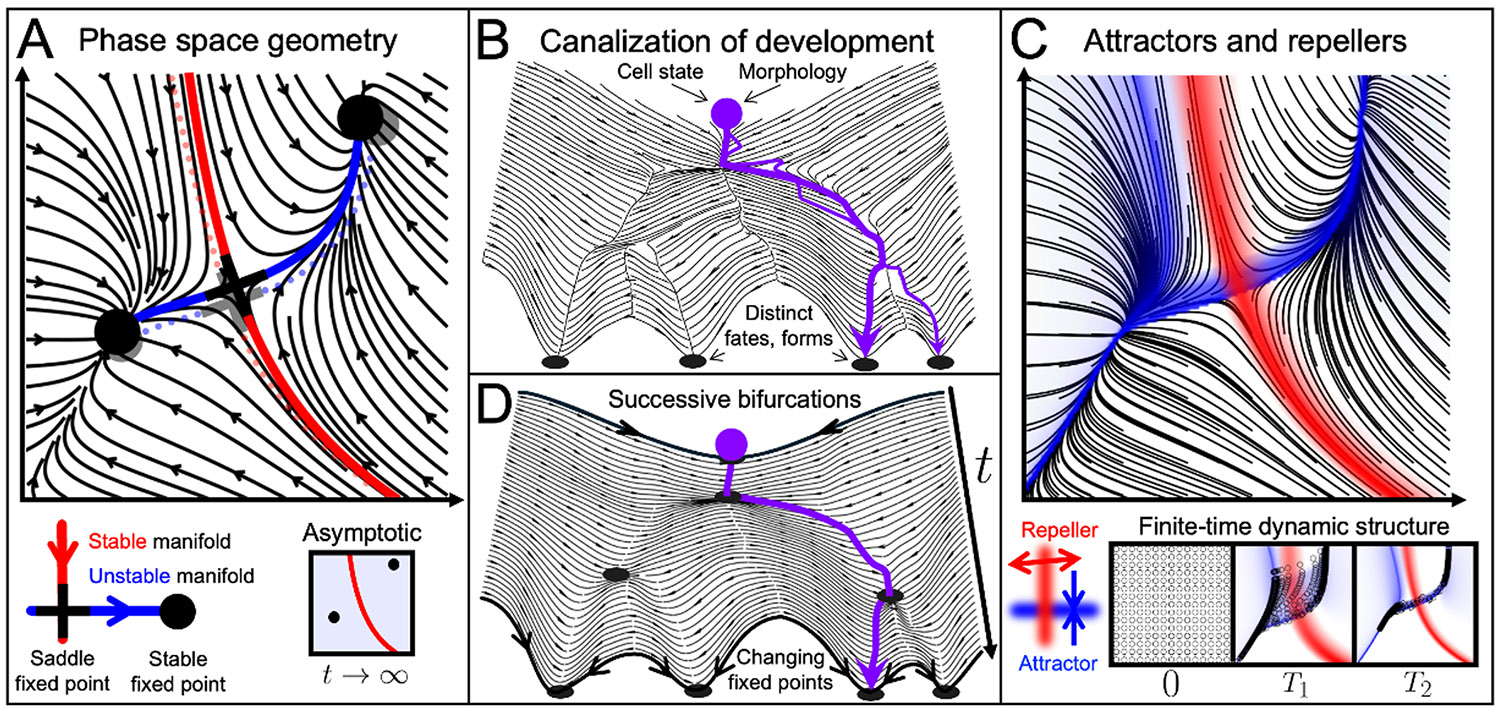
(A) Infinite-time (asymptotic) geometric structures in the phase space of a 2D autonomous dynamical system. Black lines mark trajectories, black disks mark stable fixed points, the black cross marks a saddle fixed point, and the solid red and blue curves its stable and unstable manifolds. These manifolds and fixed points constitute the geometric structure of the dynamical system, shaping its long-term (t→∞) trajectories. A small perturbation to the differential equations does not affect its geometric structure (lighter dotted curves, fixed points). (B) Waddington’s landscape (topography adapted from [19]) with streamlines (black curves). Streamlines (same as trajectories in autonomous, or time-independent, dynamical systems) depict movement down the landscape. A developmental trajectory (thick purple line)—representing cell differentiation or regulated morphogenesis—is buffered against perturbations (thin purple line), converging to the same valley unless perturbed at certain locations. (C) Finite-time geometric structures for the dynamical system shown in A. Over a finite time duration T, trajectories maximally separate from and converge towards repelling and attracting Lagrangian Coherent Structures identified from the Finite-Time-Lyapunov exponents [20,21]. These finite-time structures—distinct from their asymptotic, i.e. long-time, counterparts (panel A)—are dynamic (i.e. move), shaping trajectories over any prescribed T. The bottom panels show the dynamic of finite-time attractors and repellers for increasing $T_{1}<T_{2}$ and their influence on tracer particles (grey) initialized from a uniform grid. Attracting and repelling Lagrangian Coherent Structures are also computable for general nonautonomous (i.e. time-dependent) dynamical systems (e.g., Fig. 3 C-D), which do not have asymptotic structures as in A. (D) In a time-dependent dynamical system, the instantaneous fixed points (black dots) and streamlines (black curves)—representing trajectories of the instantaneous velocity field—change over time, typically via successive bifurcations.

For example, knowledge of a small number of fixed points—i.e., points where the state does not change—and their connectivity fully predicts the long-term behavior of large numbers of trajectories (Fig. 1A). Specifically, the complicated organization of 2D trajectories simplifies when one recognizes that they are structured around a curve connecting two stable fixed points via a saddle fixed point. In *stable fixed points* (also called attracting fixed points), all nearby trajectories converge to the fixed point, while *saddle fixed points* have one stable (red) and one unstable (blue) manifold (a curve, surface, or higher-dimensional analog in the phase space). A stable (unstable) manifold consists of all points that converge to a fixed point as t→∞(-∞). Understanding the *phase-space geometry* (or structures)—i.e. the manifolds that organize trajectories—lets us disregard each trajectory’s details while retaining their organization. For example, all trajectories starting on the right (left) of the red manifold (two *basins of attraction*) will eventually reach the right (left) stable fixed point. Additionally, these fixed points and manifolds are *structurally stable* [18]—i.e., they persist and move nearby (shown in grey) under small perturbations (e.g., from parameter uncertainties) of the dynamical system.

The initial applications of dynamical systems theory to development were motivated by the robustness and stability of developed states and developmental trajectories, which Waddington termed ‘chreodes’. The transformation from egg to embryo represents a highly nonlinear genotype-to-phenotype map [6,16,22]. Waddington identified nonlinearity as ‘canalizing’ development [23], a concept illustrated in his ‘epigenetic landscape’ [2] (Fig. 1B) and crucial to his ideas about the interplay between development and evolution [19,24]. Canalization captures the idea that developmental trajectories tend to return to similar paths when perturbed. Waddington’s landscape metaphor gained traction because it provided a tangible and visually intuitive depiction of this phenomenon in a gradient dynamical system where states flow downhill (Fig. 1B) [25]. In recent years, Waddington’s landscape is most often associated with cell differentiation. However, when introducing canalization, Waddington refers first to the canalization of the organism’s development as a whole and then separately to its constitutive processes of cell differentiation and morphogenesis [23]. Here, we consider all three and their interrelations.

### 2.1. Infinite-time vs. finite-time dynamics

In Fig. 1A, we know that trajectories will either go to the left or the right stable fixed point after infinite time (their *asymptotic* behavior). However, one might be interested in the transient behavior of trajectories [26,27] and whether distinct, dynamic geometric structures shape trajectories over intermediate (finite) times. Fig. 1C shows attracting and repelling Lagrangian Coherent Structures [20]—attractors and repellers for short—where phase-space trajectories will distinctly converge or separate over a finite time T. These attractors and repellers organize trajectories over the chosen T (Fig. 1C, bottom), revealing distinct structures compared to their asymptotic counterparts (Fig. 1A), to which they typically converge for large T (Fig. 1C, bottom). An intuitive distinction between asymptotic and finite-time structures is that the former are static in the phase space (typically anchored to a fixed point), while the latter are dynamic—regions where trajectories accumulate or separate do not need to remain fixed (see for example Fig. 3E)—and vary with T. Finite-time structures are commonly used to study geophysical flows (e.g., to identify where pollutants [28], phytoplankton [29], or people [30] accumulate in the ocean), and they are computable for any velocities or trajectories, from ocean to organoids to gene expression data. In development, attracting or repelling structures can include dynamic tissue regions or gene expression profiles where cells converge or separate.

### 2.2. Autonomous vs. nonautonomous dynamics

Another key distinction in dynamical systems is between *autonomous* dz∕dt=f(z), i.e. time-independent) and *nonautonomous* dz∕dt=f(z,t), i.e. time-dependent) systems. For example, denoting the Waddington potential function by $V(z),z=[z_{1},\ldots ,z_{n}]$, the landscape metaphor can represent either an autonomous dynamical system dz∕dt=−∇V(z), where cells move down a fixed landscape (Fig. 1B), or a nonautonomous dynamical system dz∕dt=−∇V(z,t) with the phase space z on one axis, t on another axis and new peaks and valleys emerge over time (Fig. 1D). In the former, the metaphor represents a system that evolves according to internal rules. In the latter, the metaphor represents a system that also depends on changing external parameters. Most dynamical systems theory results are for the asymptotic (i.e., as t→∞) behavior of autonomous systems—revealing geometric structures that organize trajectories over long times. These include fixed points, limit cycles, and their associated stable and unstable manifolds [31], as in Fig. 1A. However, these results do not hold for nonautonomous dynamical systems [31]. Even in autonomous systems, the relevant geometric structures governing trajectories over short [30,32,33] and finite times [20] differ from their asymptotic counterparts. A key advantage of finite-time attractors and repellers (Fig. 1C) is that they are computable for both autonomous and nonautonomous systems, which no longer have structures like fixed points or their stable and unstable manifolds (Fig. 1A). Furthermore, attractors and repellers can also be computed in dynamical systems defined by experimental data instead of equations.

### 2.3. Global vs. local embryo models

Landscapes remain an appealing metaphor for organizing observed phenotypes—represented by adjacent basins—in natural [16] and stem-cell based embryo [34-36] models. Global models of whole-embryo dynamics (i.e., from egg to developed embryo) may account for development’s constrained responses to environmental and genetic perturbations, including phenocopying, dual buffering, and global epistasis, reviewed in [37]. While appealing, the whole-embryo dynamical systems concept is challenging to operationalize. Distinct phenotypes and their responses to different conditions may be clear, but the underlying dynamical system remains ambiguous or entirely abstract, precluding quantitative predictions.

**Fig. 2.**
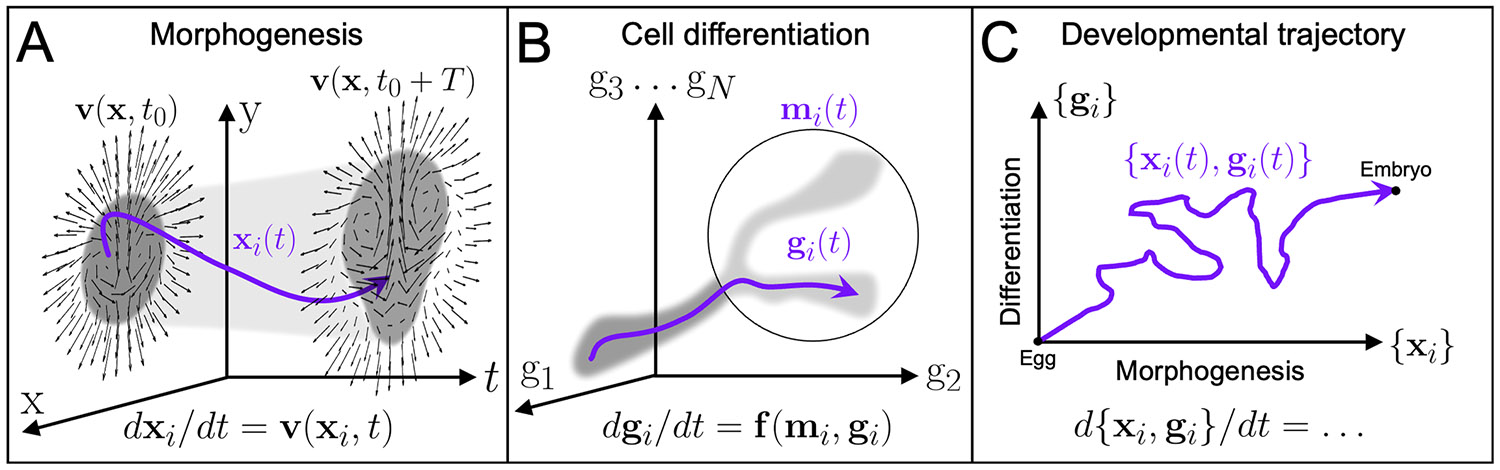
(A) Non-autonomous dynamical system describes morphogenetic movements $\left\{x_{i}(t)\right\}$ during avian gastrulation. Purple curve traces one cell’s spatial trajectory $x_{i}(t)$ and v(x,t) shows the embryo-scale velocity field (adapted from [41]) at an initial ($t_{0}$) and later ($t_{0}+T$) time. Grey clouds represent embryonic cell positions $\left\{x_{i}\right\}$ at each time. (B) Gene expression space. Grey cloud represents gene space trajectories of a population of cells $\left\{g_{i}(t)\right\}$. Purple curve traces one cell’s gene expression trajectory $g_{i}(t)$, passing through a decision region (circled) and influenced by external signals $m_{i}(t)$—e.g. molecular morphogens or mechanics. (C) Embryo developmental trajectory, including cells’ positions $\left\{x_{i}(t)\right\}$ (morphogenesis) and gene expression $\left\{g_{i}(t)\right\}$ (differentiation).

Here, we focus on ‘local’ embryo models, deconstructing the high-dimensional spatiotemporal data collected on embryos during specific developmental stages. The embryo exhibits organization across molecular, cellular, and multicellular scales. Abstracting above most molecular-scale details, one can describe the embryo’s state as the union of transcriptional states and positions for each constituent cell i. Changing cell numbers complicates this description but does not affect the compressed representations considered here. The embryo’s morphological state comes from the collection of cell positions $\left\{x_{i}\right\}$ in 3D position space x=[x,y,z] (Fig. 2A), while the transcriptional states collectively patterning the morphological state can be represented by $\left\{g_{i}\right\}$, where $g_{i}$ is a vector of thousands of gene expression levels characterizing the ‘gene space’ position of cell i (Fig. 2B). We denote vector quantities associated with cell i by $z_{i}$, and brackets $\left\{z_{i}\right\}$ denote the union of these vectors for all cells considered. Before any dimensionality reduction, the embryo’s state is $\{x_{i}$, $g_{i}\}$. Resolving or reconstructing trajectories {$x_{i}(t)$, $g_{i}(t)$} (Fig. 2C) is the aim of live imaging [38], lineage tracing—including recent genomic and CRISPR-based barcoding methods [11]—spatial transcriptomics [39], and trajectory inference techniques [40].

It is practical to partition development into local spatial and temporal processes—specifically, a sequence of developmental stages with associated morphological transformations and cell fate decisions. Developmental stages typically demarcate the start or cessation of some orderly morphological movements [42], i.e. transformations in $\left\{x_{i}(t)\right\}$ (Fig. 2A). Fate decisions, instead, reflect diverging trajectories $\left\{g_{i}(t)\right\}$ among alternative cell fates—point clouds in gene space—with highly constrained transitions between them [43] (Fig. 2B). In each specific stage and decision, it is common to consider a simpler, tailored dynamical system with few parameters, enabling significant dimensionality reduction [18].

In each local model, identifying the appropriate class of dynamical systems, phase space variables and parameters is crucial. For example, the differentiation of a cell exposed to a constant signaling environment ($m_{i}$) can be described by an autonomous dynamical system ($dg_{i}(t)∕dt=f(g_{i},\;m_{i})$) and its asymptotic structures. In contrast, morphogenetic movements are typically described by nonautonomous dynamical systems $dx_{i}(t)∕dt=v(x_{i},\;t)$ where velocity fields are explicitly time dependent—i.e. changing in time at fixed spatial locations (Fig. 2A). In the following sections, we review local dynamical systems models for cell fate decisions ($g_{i}$), gene expression patterning ($\left\{g_{i}\right\}$), and morphogenetic movements ($\left\{x_{i}\right\}$), emphasizing the geometry of their phase space structures (as in Fig. 1). Finally, we discuss how local models might be integrated to understand processes where patterning and morphogenesis are inextricable due to self-organizing feedback.

## 3. Fate decisions

There are several techniques to reconstruct gene-space trajectories $g_{i}(t)$ [44-46]. While crucial for characterizing diverging gene expression profiles and extracting their skeletons [47], these approaches are descriptive/kinematic. They identify expression trajectories and decision regions (Fig Fig 2B, circle), but not how decisions are made, which may depend on other internal variables like chromatin organization [48], metabolic activity [49], or external parameters $m_{i}$ not explicitly represented in gene space [4,17]. These parameters may include a cell’s mechanical environment or molecular morphogens, whose spatiotemporal dynamics coordinate fate decisions [50]. Inferring dynamics from single-cell data often relies on a steady-state assumption [51] that may not be justified when signals are time-dependent. Understanding cell differentiation requires learning how $m_{i}(t)$ interacts with internal states to affect $g_{i}(t)$.

### 3.1. Gene regulatory networks

Given external signals $m_{i}$, gene-space trajectories depend on the dynamics of a cell’s internal state $g_{i}$, commonly represented as interacting networks of genes called gene regulatory networks (GRNs) [52, 53]. Kauffman pioneered the application of dynamical systems theory to GRNs, initially using Boolean networks, characterizing cell types via discrete dynamical systems with each gene on ($g_{i}=1$) or off ($g_{i}=0$), and later as fixed points of continuous dynamical systems where $g_{i}\geq 0$ [43,54,55]. The dynamical systems perspective led to the identification of discrete attractors in gene space corresponding to stable cell types [56] and bifurcation events [57]. While GRNs capture intracellular dynamics, they require extensive kinetic parameters [58] for which measurements are limited [59]. However, for understanding cell fate decisions—the transitions between discrete cell types—many GRN details may be unnecessary.

Alternatives to this bottom-up approach include top-down approaches focused on cell fate decision dynamics. In [60], Rand, Corson, Siggia, and coauthors cast the gene regulatory dynamics near decision regions as gradient-like Morse-Smale systems—structurally stable systems where trajectories flow towards stable fixed points without forming closed cycles. While this classification only approximates GRN complexity and may only hold in decision regions, it has been justified by three phenomenological features of cell differentiation already recognized by Waddington [2,19]: *i)* a finite number of stable cell types, *ii)* directional, canalized fate transitions (Fig. 1B), and *iii)* system robustness. Near decision regions (Fig. 2B), the phase space geometry structures the dynamics, similar to the illustration in Fig. 1A, even without knowing the underlying GRN. Assuming that cell types correspond to nearby stable fixed points in a region of gene space, [60] shows that Morse-Smale systems allow limited stable fixed points configurations (connected by saddles’ unstable manifolds) and bifurcations (via changing $m_{i}$) to cause transitions between them. These asymptotic phase space structures provide the ‘geometry’ (or skeleton) of decision-making processes. Decision regions in gene space are high dimensional, yet the configurations and connectivity of their few stable fixed points remains enumerable.

### 3.2. Decision spaces

Recent ‘geometrical’ or *gene-free* models use the aforementioned frameworks to reduce the dimensionality of fate decisions to their theoretical minimum [60-65]. See [66] and [9] for detailed reviews. For the ubiquitous motif of a precursor cell deciding between two alternative fates, instructed by up to two external parameters, [60] establishes that all possible geometries can be modeled with a two-dimensional dynamical system. In these models, gene-space stable fixed points correspond to equivalent fixed points in a two-dimensional ‘decision space’ [66], whose axes do not need to correspond to specific genes [15], though a recent study seeks explicit connections [67].

**Fig. 3.**
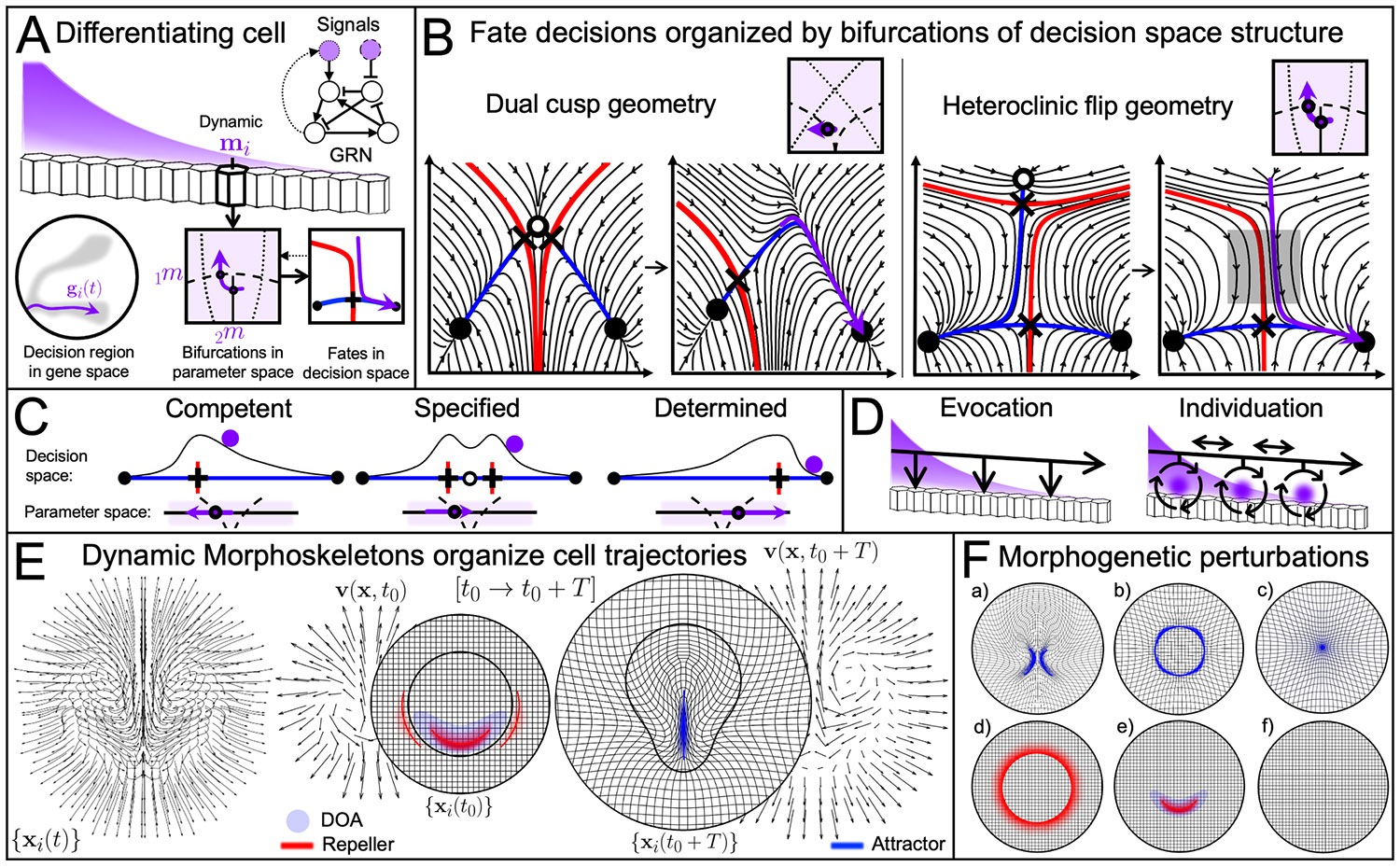
(A) Gene regulatory network (GRN) dynamics respond to external signals (purple), such as local morphogen concentration $m_{i}$—at the position $x_{i}$ of cell i. In gene-free fate decision models, up to two external signals (mi=[m1,m2]i) act as control parameters, tilting a decision landscape that encodes the organizing structures of a decision region in gene space. Most parameter changes distort the landscape without affecting fixed points or their connectivity, leading to similar (or topologically equivalent) dynamics. However, when parameters cross bifurcation curves (dashed and dotted curves) in parameter space, bifurcations create, destroy, or alter the connectivity of fixed points, affecting fate decisions. (B) Two alternative decision geometries (from [61]) governing the transition from a precursor cell type (white disk) to two alternative fates (stable fixed points, black disks), separated by saddles (black crosses). In the *dual cusp* geometry, named for the cuspoidal point where the dashed curves meet, a fold (local) bifurcation (crossing a dashed curve) annihilates the right saddle and precursor fixed point, leaving the cell in the basin of attraction of the right stable fixed point. An alternative geometry involves a *heteroclinic flip* bifurcation (global, crossing solid curve) where the unstable manifold (blue) of the upper saddle flips its stable fixed point connection from left to right. A subsequent fold bifurcation (local, crossing dashed curve) annihilates the upper saddle and precursor fixed point, directing a transition to the right fate. Cells exiting the precursor state remain near the lower saddle’s stable manifold (red), transiently amplifying sensitivity (boxed region) to perturbations as it flows to the right stable fixed point. (C) Cell fate commitment concepts with respect to a range of changes to one parameter. A competent cell can be induced to descend a landscape in decision space (as in B, left). It becomes specified when it remains committed after signal removal (middle), and determined when no signal can alter its commitment. (D) Waddington’s *evocation*—the initiation of a developmental response by an external signal—and *individuation*—elaboration via cell-cell interactions, introduced in his work on organizers [2]. (E) *Dynamic Morphoskeleton* (DM) [21] during avian gastrulation. Cell trajectories $x_{i}(t)$ (from [41]) over [$t_{0}$, $t_{0}+T$] from $t_{0}\approx$ HH1 stage to HH3 stage (T≈12h). Outer cells spread radially, while inner cells exhibit polonaise movements and convergent extension. The DM consists of repellers and attractors concisely organizing cell trajectory data (compare with Fig. 1C): repellers (red) are dynamic curves where cells maximally separate, shown on the tissue’s undeformed Lagrangian coordinates $x_{i}(t_{0})$, while attractors (blue) are dynamic curves where cells maximally converge, shown on the deformed configuration $x_{i}(t_{0}+T)$. Mapping an attractor back to $x_{i}(t_{0})$ reveals its domain of attraction (DOA), the region that converges to it during [$t_{0}$, $t_{0}+T$]. (F) Perturbing control parameters (e.g., cell behaviors and initial or boundary conditions) produces alternative DMs in mechanochemical models and experiments. Top: varying initial conditions and cell ingression can yield twin attractors (a), a ring attractor (b), or a point attractor (c) [70,71]. Bottom: The repellers can be independently eliminated by blocking active cell intercalations (d), edge cell crawling (e), or both (f) [41].

Gene-free models typically consider nonautonomous dynamical systems, with a 1D [68] or 2D phase space [15,61-64,69] and use asymptotic methods to analyze the dynamics for given control parameters $m_{i}$ (Fig. 3A). The nonautonomous behavior comes from time-dependent control parameters ($m_{i}(t)$) uniformly tilting the landscape to create and destroy fixed points (local bifurcations) or change the connectivity between existing fixed points (global bifurcations) shown in Fig. 3B [60]. Bifurcations are changes in the phase space geometric structures—i.e., the existence and connectivity of fixed points—as control parameters cross bifurcation boundaries. Bifurcation diagrams draw these boundaries (curves or surfaces) in parameter space, providing a mapping between parameter regions and alternative, structurally stable phase space structures (Fig. 3A). While dynamic control parameters could be included in an autonomous dynamical system by increasing the phase space dimension, they typically represent extracellular signals, justifying bifurcation analysis to study how their changes affect intracellular dynamics. Dependence on control parameters also gives non-autonomous models the flexibility to predict phenomena like fate conversion or reversal in addition to the unidirectional decision-making of a static landscape.

Gene-free models start by identifying the involved cell types, which is nontrivial, as it requires partitioning the gene space and may further depend on chromatin accessibility and other modalities absent from transcriptomic data [72]. Even when cell types are known a priori, modeling typically requires thresholding continuous data to infer transitions [15,47,61]. Among possible arrangements of phase space geometries on bifurcation diagrams, some are ruled out for requiring parameter fine-tuning [9,15,61]. Others differ in dimensionality: in effectively 1D geometries, fate conversion requires passing back through the precursor type via a local bifurcation (fold, Fig. 3B, left), where a stable and saddle fixed point are mutually created or annihilated, whereas intrinsically 2D geometries may allow direct conversion between the alternative fates [9,61], e.g. via a global bifurcation (heteroclinic flip, Fig. 3B, right), where a saddle fixed point suddenly connects to a different stable fixed point. The possible bifurcations are also constrained by the number of control parameters—e.g., just one needed for folds, two for heteroclinic flips [60]. Each of these differences has implications for experimental predictions. Including noise in gene-free models allows cells to fluctuate between the basins of attraction [15]. Fate decisions are not purely deterministic, and stochasticity can facilitate correct fate proportions [64,73]. In a dynamical system, noise can be extrinsic (fluctuating control parameters) or intrinsic (phase space variables fluctuations) [74] and additive (independent of a cell’s state) or multiplicative (state-dependent). Intrinsic, multiplicative noise, common in GRNs, may also stabilize, destabilize, or reposition fixed points, shaping the effective landscape [75].

Gene-free models predict fate decisions based on an initial condition, noise terms, and extracellular signals $m_{i}(t)$—tilting the landscape to induce bifurcations (Fig. 3A-B). Given a candidate phase space geometry, Bayesian methods [15,62,63,69] or neural networks [67] can learn this tilting (i.e., the bifurcation diagram) by minimizing errors in fate prediction and time-course statistics from experimental data. Fitting the data to alternative geometries facilitates comparison of their predictive power but requires experimental manipulability of $m_{i}(t)$. Experimental perturbations include modifying morphogen levels, feedback via ligand production, signal transduction [64], signaling gene expression [62], or—if modeling interacting cells—signaling ranges [68]. Dynamic perturbations like morphogen pulses have proven especially informative for discriminating between alternative geometries [9,61]. For example, in the mouse blastocyst, a bifurcation diagram with a dual cusp (Fig. 3B, left) predicts monotonic fate commitment, meaning that FGF morphogen pulses become less effective for fate conversion over time. In contrast, a bifurcation diagram with a heteroclinic flip (Fig. 3B, right) predicts an intermediate time of maximal sensitivity to perturbations (gray square), facilitating finer control over fate proportions [61].

While gene-free models use asymptotic methods (see Sec. 1), fate decisions may occur during limited competence windows, and there is a finite time between elimination or escape from a precursor stable fixed point and specification or determination at a new one [61]. If signals change at rates comparable to fate decisions, the rate at which bifurcation points are crossed may become important [76]. Additionally, new phenomena can arise, even in simple, structurally stable systems. [77] classifies non-autonomous phenomena like *captures*—where a trajectory is drawn into a moving stable manifold—and *pursuits*—where it follows but never reaches a moving instantaneous fixed point—both arising from relative motion between trajectories and time-dependent fixed points or their manifolds. Finite-time attracting and repelling structures (Fig. 1C)—complementary to asymptotic structures (Fig. 1A)—are appropriate tools to tackle these scenarios and may provide insights into transitory behavior.

Control parameters $m_{i}(t)$ can be either prescribed [15] or coupled to $g_{i}(t)$. For example, in a differentiation model in the early mouse blastocyst, a prescribed control parameter represents a shrinking competence window, forcing a fate decision [61]. In an *in vitro* model of mouse embryonic stem cell (mESC) differentiation, endogenous signaling (effect of $g_{i}$ on $m_{i}$, dotted arrows in Fig. 3A) is inhibited so that morphogen signals could be controlled externally [15]. Gene-free modeling of *C. Elegans* vulval development instead emphasizes feedback, with cells exposed to a combination of prescribed EGF signaling and endogenous autocrine and paracrine Notch signaling, activated when a cell crosses a threshold in decision space [62-64,68,69]. Such endogenous signaling can be necessary to predict correct fate patterns or proportions [61,68]. Theoretically, feedback between $g_{i}$ and $m_{i}$ may be optimal for achieving accurate spatial patterning [78], but this feedback implies that some control parameters are not entirely external and could be treated as phase space variables. Nevertheless, separating and manipulating morphogens as external control parameters elucidates their role in individual fate decisions.

Beyond elucidating the geometry of fate decisions, gene-free models offer concise, intuitive explanations for classical embryology concepts [60,62,69] (Fig. 3C). *Competence* depends on susceptibility to receive a signal (e.g., via receptors) and change stable fixed points [2]. A cell is *specified* if it remains committed to the same fate after signal removal, occupying the same basin of attraction. It is *determined* if it irreversibly remains in a basin of attraction, even when exposed to signals that could previously have changed it [1]. Gene-free models also integrate the effects of changing multiple external signals—linearly combining to tilt the landscape—with all non-linearities captured in the landscape itself [62,67].

## 4. Static tissue patterning

The extracellular signals $m_{i}(t)$ (supplied to cell i) form imposed or self-organizing patterns in space that are inputs for intracellular fate decisions. In this section, we consider tissue patterning where cells remain fixed in space ($\left\{x_{i}(t)\}=\{x_{i}\right\}$). If signals $\left\{m_{i}(t)\right\}$ are relatively uniform or well-mixed, different precursor cells in the same decision region may share a common landscape [61]. In contrast, heterogeneous morphogen patterns entail different landscapes for differently positioned cells. If $m_{i}$ differs between cells and depends on $g_{i}$ and cell-cell interactions, then the integrated system dynamics $g_{i}(t)$, $m_{i}(t)$ is needed and fate decisions cannot be reduced to independent cell dynamical systems for $g_{i}(t)$. These cases constitute self-organized patterning—what Waddington termed *individuation*—in contrast to mosaic patterning or initial patterning by unidirectional provision of instructive or permissive signals—what Waddington termed *evocation* [2] (Fig. 3D). Patterning models for $g_{i}(t)$, $m_{i}(t)$ rapidly grow complex as each cell adds new dimensions to the phase space. Therefore, geometric approaches to patterning have focused on a small number of discrete, interacting cells or continuum models. Cell-scale fate pattern models are suitable in *C. Elegans* vulval development [62-64,69] because the few accessible fate patterns fit on a phase diagram parametrized by global morphogen signaling. Similarly, in *Drosophila* bristle patterning, a small set of cells interact through a distance-dependent coupling, with individual fate decisions governed by a fold bifurcation, refining a coarse prepattern into finer stripes (just a few cells wide) [68]. For less precise patterning involving more cells, continuum models have advantages, using partial differential equations to describe reaction-diffusion dynamics for a continuum of cells ($\{x_{i}\}\to x$). In addition to initial conditions, continuum spatial models require boundary conditions, constraining what happens at spatial domain boundaries. While they are mathematically infinite-dimensional, analytical methods, including linear stability analysis, provide insights into the geometry of spatial patterning. These models do not always distinguish between morphogen pattern formation and interpretation, with gene expression and morphogen dynamics lumped into the same variables. Typically, reactions represent g(x) and feedback with m(x) while diffusion represents cell-cell interactions.

Turing’s identification of instabilities in reaction-diffusion systems remains an influential framework for continuum patterning, providing conditions for self-organized pattern formation that do not depend on specific molecular details [79,80]. Varying the effective reaction kinetics and diffusion parameters in a spatially-extended system with a generic ‘activator’ and ‘inhibitor’ can induce bifurcations from uniform states to spatially patterned states [81]. The gene-free dimensionality-reduction approach has been extended to Turing systems by fitting their spectral dynamics to a gradient flow landscape [9,60]. Patterns’ bifurcations can depend on choices of boundary conditions [82] and—as with individual fate decisions—stochasticity can shift the bifurcation boundaries [83]. Turing systems are just a subset of pattern-forming systems [84-86]. Other reaction-diffusion models include oscillatory dynamics, crucial in developmental processes like the vertebrate segmentation clock (reviewed in [87]) and signaling waves in excitable and oscillatory systems (reviewed in [88]). Bifurcations involving stable limit cycles—periodic orbits attracting nearby trajectories—can translate time periodicity into periodic spatial patterns [89,90].

Complementary to reaction-diffusion models [85], Wolpert’s positional information paradigm emphasizes directional flows of information from instructive morphogen patterns to fate patterns [91,92]. By analyzing mutual information between $g_{i}(t)$ (or $m_{i}(t)$) and $x_{i}(t)$ (relative to a landmark), positional information draws special attention to patterning precision and canalization [93,94]. For example, scaling a pattern with variable embryo length L requires $\left\{g_{i}\right\}$ to have more mutual information with $\left\{x_{i}\right\}∕L$ than $\left\{x_{i}\right\}$ [95]. In *Drosophila*, a maternal morphogen gradient (time-varying control parameter [96]) does not scale with L, whereas downstream gene expression is scale-invariant [95]. For reaction-diffusion models, this information-theoretic observation has a geometric implication for the dynamics: a 1D manifold of neutrally stable fixed points for proportional expression patterns g(x∕L) along the axis of embryos of different lengths. This requires directions in phase space along which fluctuations neither linearly grow nor decay [95]—consistent with experimental observations [97]. This illustrates a link between high-dimensional phase space structures and spatial pattern canalization. Because noise can significantly—and constructively—affect pattern formation, explicit consideration of stochastic dynamical systems can provide insights into robustness, precision, information transmission, and error-correction mechanisms in development [92,94].

## 5. Morphogenetic movements

Tissue configurations $\left\{x_{i}\right\}$ are typically dynamic during development. Before reviewing how $\left\{x_{i}(t)\right\}$ and $\left\{g_{i}(t)\right\}$ trajectories interact in dynamic tissue patterning, we review the historical development and recent advances in dynamical systems approaches to morphogenetic movements $\left\{x_{i}(t)\right\}$.

### 5.1. Morphometrics to morphogenesis

Morphometrics quantify an embryo’s form, measuring lengths, length ratios, distances between morphological landmarks and statistical properties of embryo outlines to compare morphology within or between species [98]. Morphometrics are the axes for low-dimensional ‘morphospaces’ [99] and are frequently accompanied by mechanochemical models [100-102] to map mechanistic parameters to phenotypes. Typically, however, morphometrics quantify embryos’ final, static form rather than their dynamic form during development [42, 103]. Understanding of development is concerned not just with form but formation—morphogenesis over morphology.

Pere Alberch advanced a dynamical perspective by adding a time axis to traditional morphospaces, describing developmental trajectories that he viewed as analogous to Waddington’s chreods [42,104]. Alberch emphasized that while morphogenesis is continuous, the onset (at $t_{0}$) and cessation (at $t_{0}+T$) of different movements during distinct time intervals [$t_{0}$, $t_{0}+T$] demands morphogenetic models tailored to each developmental stage [42], as with local models for individual fate decisions. This approach brought dynamical systems considerations to the fore of evo-devo, placing concepts like heterochrony, evolvability, and developmental constraints in a dynamical systems context [22,105,106]. While trajectories in simplified morphometric coordinates capture coarse morphological changes (e.g., size and shape), they typically neglect structured interior rearrangement (deformation). However, the changing outline of a form can be associated with a wide variety of deformation that represents the more proximal consequences of tissue mechanics.

### 5.2. Thompson to Thom

In his seminal work *On Growth and Form* [3], D’Arcy Thompson introduces coordinate transformations as a method to compare both overall shape and internal spatial relationships. While he emphasized transformations between adult forms, he also considered interpolated coordinates representing transitional forms. The same approach can be applied to development, mapping transformations between morphologies in consecutive developmental stages [107]. In illustrations of deforming Cartesian grids, the order of lines never crosses [3] (e.g., see Fig. 3E), representing smooth transformations. Thompson acknowledged that some morphologies exhibit discontinuities in their morphological organization and, at the cell scale, neighbor exchanges and random motility disrupt this neat positional order. However, at the tissue or embryo scale, the morphogenetic movements within a developmental stage often exhibit smooth deformation through growth, stretching, or convergent extension.

Where Thompson leveraged continuity, the mathematician René Thom emphasized discontinuity. For Thom, morphogenesis consisted of approximately continuous transformations punctuated by abrupt, topological changes—e.g., in gastrulation or neural tube closure—that he sought to understand in terms of structurally stable dynamical systems and bifurcations [18,108]. Thom had an ambitious and analytically intensive view of morphogenesis, but, in contrast to his lasting influence bringing catastrophe theory into biology [16,60], struggled to ground his more speculative morphogenetic models in the available data. In the era of live imaging, cells’ spatial trajectories $\left\{x_{i}(t)\right\}$ can now be extracted and analyzed in many model systems, and new fluid and nonlinear dynamics techniques are well suited to analyze morphogenetic flows.

### 5.3. Eulerian (lab) vs. Lagrangian (cell) frames

**Fig. 4.**
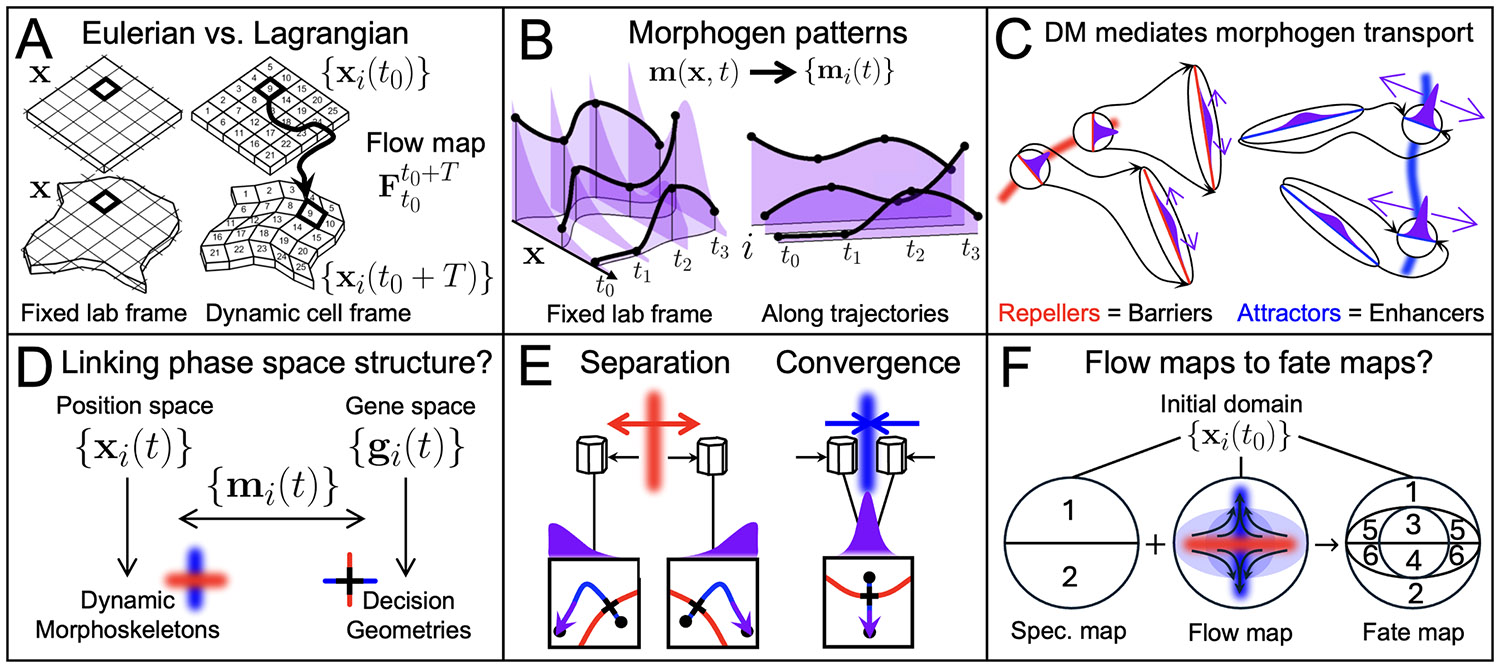
(A) Eulerian coordinates mark fixed positions in space (the lab frame, left) through which cells and morphogens move. Lagrangian coordinates (the cell frame, right) label cell identities i (or the initial position of a tissue patch) and follow cells along their trajectories represented by the flow map $F_{t_{0}}^{t_{0}+T}(x_{i}(t_{0}))$. (B) Eulerian morphogen concentrations m(x,t) (left, purple) in 1D over time with cell trajectories (black curves) moving through them (height reflects morphogen levels). The morphogen exposure of each cell i depends on its trajectory $x_{i}(t)$. $\left\{m_{i}(t)\}(\neq m(x,t)\right)$ are the time-dependent control parameters for cell fate decisions. (C) The DM mediates cells’ dynamic interaction ranges [112]. Repellers (attractors) in morphogenetic flows reduce (enhance) morphogen diffusive fluxes by flattening (sharpening) morphogen gradients in the cell frame through sharp cell separation (convergence). (D) Complex trajectories in position and gene spaces (c.f. Fig Fig 2) compressed and organized by low-dimensional Dynamic Morphoskeletons and decision geometries (c.f. Fig Fig 3). The DM can mediate intercellular communication via morphogens $\left\{m_{i}(t)\right\}$ and influence fate decisions. (E) Repellers, reducing cell-cell communication, support fate bifurcation by compartmentalizing cells’ signaling environments. Attractors, enhancing cell-cell communication, homogenize cells’ signaling environments supporting common fate induction. (F) Sketch of two initial regions (1 and 2 on a specification map) giving rise to six (1–6 on a fate map) due to the interplay between signaling and morphogenetic constraints. Black arrows depict representative cell trajectories. The blue circle and ellipse represent consecutive domains of attraction over a shorter and longer time intervals beginning at $t_{0}$. Cells in the wider domain differentiate but acquire different fates because they reach the attractor at different times (3 vs. 5, 4 vs. 6). As cells converge horizontally, a repeller separates them vertically, constraining communication between 1 and 2 to bias differentiation into odd or even fates.

As in classic fluid flows, Eulerian quantities are common in visualizing morphogenetic flows. These include time-dependent velocities and their spatial gradients (vorticity and strain rates) in the lab frame—i.e. fixed Eulerian grid positions x (e.g. v(x,t) in Fig. 3E). In classic fluids, Eulerian coordinates are the most appropriate because there is no reference or equilibrium configuration or a need to follow individual fluid elements. However, in morphogenesis, the focus is on understanding the motion of single cells $x_{i}(t)$, the relative motion of nearby cells $x_{i}(t)-x_{j}(t)$ (or the deformation of tissue patches) during a developmental stage of interest between time $t_{0}$ and $t_{0}+T$. In this context, Lagrangian coordinates—labeling space by initial cell positions $x_{i}(t_{0})$ (or initial positions of tissue patches) and following their paths—are the most natural (Fig. 4A). When the goal is to identify velocity patterns [109,110] or their temporal changes [111], rather than cumulative deformation, Eulerian quantities are appropriate.

Lagrangian coordinates—implicit in Thompson’s deforming Cartesian grids—track time-dependent morphogenetic transformations relative to an initial reference configuration $\left\{x_{i}(t_{0})\right\}$ via the *flow map* $F_{t_{0}}^{t}(x_{i}(t_{0}))=x_{i}(t)$, a concept from dynamical systems. $F_{t_{0}}^{t}(x_{i}(t_{0}))$ encodes trajectories of specific tissue patches or cells, resonating with classical fate maps, which label tissue regions by their final destinations and associated fates. Lagrangian approaches to analyze tissue flows rely on the flow map and its spatial derivatives, computable from experimental velocity fields or segmented cell trajectories. Recently, Lagrangian approaches have proven useful for characterizing cumulative material transformations associated with different developmental stages [113], including *avian* gastrulation [21,114,115], axis elongation [116], primitive streak regression [117], and heart tube formation [118]; *drosophila* gastrulation [21,119,120], pupal wing [121] and wing disc pouch eversion [122]; and *zebrafish* trunk neuroectoderm convergence [119], axis elongation [12], gastrulation, and early segmentation [123]. With a Lagrangian description of morphogenesis, the question remains: What robust geometric structures—if any—organize cell trajectories?

### 5.4. Dynamic Morphoskeletons

A geometric perspective on morphogenesis should account for two key features of morphogenetic movements: *i) Material coherence*—multicellular systems move and deform collectively in a reproducible, structured manner, distinct from chaotic or random flows, and *ii) Non-autonomous* dynamics $dx_{i}∕dt=v(x_{i},\;t)$—velocities are time dependent. Hence, their instantaneous streamlines (e.g. Fig. 1A) no longer reveal motion (trajectories) [20,32]. Material coherence requires *objective* (frame-invariant) quantities to distinguish physical tissue deformation, which does not depend on the arbitrary choice of the reference system used to describe motion, from frame-dependent drifts or rotations of the whole embryo. The non-autonomous nature of morphogenesis and their characterization via finite-time datasets instead of equations precludes asymptotic (T→∞) analysis and also limits the utility of typical instantaneous (T→0) measures (e.g., velocities or streamlines), which are frame dependent [32]. While there are objective Eulerian measures of short-time material coherence [32, 33], they remain suboptimal to quantify cumulative process (Fig. 2A).

Unlike $g_{i}(t)$, positional dynamics often includes global drifts and rotations (of the embryo), which appear in measured v and $x_{i}(t)$, but do not reflect morphogenesis or its mechanisms. These confounding artifacts—typically time-dependent—are challenging to remove manually but are resolved automatically by objective Lagrangian quantities. Lagrangian Coherent Structures (LCSs) [20] identify the objective geometric skeleton of dynamical systems trajectories for any desired time interval [$t_{0}$, $t_{0}+T$] in autonomous and nonautonomous dynamical systems. There are multiple kinds of LCSs capturing distinct types of coherence [20], originally devised for complex fluid flows in earth and atmospheric sciences. The relevant LCSs for morphogenetic movements include *attractors*, where cells maximally converge starting from their *domain of attraction*, and *repellers*, where cells maximally separate during an interval [$t_{0}$, $t_{0}+T$].

Attractors, domains of attractions, and repellers constitute the *Dynamic Morphoskeleton (DM)* [21]. Attractors and repellers are dynamic geometric structures in the phase space—to be contrasted with the static asymptotic structures of autonomous dynamical systems (Fig. 1A)—that can be identified as ridges (or high values) of finite-time Lyapunov exponent (FTLE) fields (Fig. 1C), defined from the largest singular values (i.e. scalar fields) of the flow map gradient [21]. The DM compresses and organizes complex cell trajectory data $\left\{x_{i}(t)\right\}$ (Fig. 3E, left) into a few geometric structures (Fig. 3E, right). For example, in avian gastrulation, the DM consists of one attractor—the primitive streak; its domain of attraction—marking the initial positions of cells that will converge to the attractor; and two repellers. One bisects the domain of attraction (i. e., the mesoderm internalizing region) into an anterior and posterior domain, and another on the boundary between embryonic and extraembryonic tissues (Fig. 3E). Therefore, the DM pinpoints evolving boundaries between regions of different dynamics, which are inherently Lagrangian, providing a geometric framework to study $\left\{x_{i}(t)\right\}$ analogous to the one structuring gene space trajectories (Fig. 3A-B). Public algorithms enable computing the DM from confluent tissue flows [21] and sparse and noisy trajectories [124]. In recent years, DMs have been identified for many developmental stages, including chick gastrulation stages HH1-HH3 [21,41,70] and HH4-HH6 [117],Drosophila gastrulation [21], and zebrafish axis elongation [12]. Beyond compressing complex 4D cell motion data, owing to its frame-invariance property, the DM is advantageous for comparing morphogenetic processes.

### 5.5. Mechanisms of morphogenesis

Analogous to how a given fate decision geometry can be generated by many GRNs [9], DMs can be generated by many heterogeneous forces or cell behaviors. Once identified, DMs provide low-dimensional, robust objects to design targeted experiments [12,117] and scaffold a mechanistic understanding of morphogenesis. Similarly, when biophysical assumptions are built into computational models of cells or tissues, the DM can validate the robust geometric structures organizing model trajectories, filtering out their unnecessary details. Models of morphogenesis can be discrete—modeling individual cells’ dynamics—or continuous—modeling tissue deformations. Discrete models (e.g., vertex [125,126] or active tension network [127,128] models) are explicitly Lagrangian. These can be calibrated by cell-scale measurements and are useful for understanding the local integration of cell behaviors into tissue-scale phenomena [129]. However, they typically require many parameters and cannot easily reproduce embryo-scale tissue flows.

Continuum models, instead, lose direct connection to precise cell behaviors but have few parameters and successfully reproduce embryo-scale tissue flows [130,131]. Mechanochemical continuum models reproduce chick gastrulation flows and their DM (Fig. 3E) [41,70]. Additionally, they enable deconstructing the mechanisms causing the DM. By varying a parameter associated with cell ingression and the initial actomyosin-generated active stress distribution, the line-shaped attractor can be eliminated or reshaped into a ring- or point-shaped attractor [70] (Fig. 3F, top). Consistent with model predictions, experimental manipulations in living chick embryos resulted in morphogenetic movements and DMs reminiscent of gastrulation flows naturally observed in teleost fish, amphibians, and reptiles [71,132]. Mechanochemical modeling also identified separate mechanisms responsible for each repeller, enabling their independent elimination (Fig. 3F, bottom) in the model and experiments [41]. The modular controllability of repellers and their independent generating mechanisms suggest separate mechanisms the embryo employs to control its dynamic size and shape [41].

Besides controlling attractors and repellers [41,70,133], this approach can be used to investigate canalization and bifurcation of morphogenetic movements. For example, an active stress initial condition with distinct mesendoderm precursor regions can generate twin attractors (Fig. 3F, top), resulting in a second body axis, as occasionally observed in experiments [70]. Decreasing the distance between the bumps causes a discontinuous change from two attractors to one. Similarly, mechanical boundary conditions—constraining tension propagation through the epiblast—have been shown to affect primitive steak formation [134], so that cut fragments of the chick epiblast each form a body axis (attractor) [135], a prime example of tissue-scale canalization in embryogenesis.

## 6. Unifying morphogenesis and differentiation

Decision geometries and DMs organize cell fate dynamics and positional trajectories in their respective phase spaces. Both provide insights that do not depend on specific molecular or mechanical details. Mechanistic models for fate patterning are often molecular, involving GRNs and morphogen control parameters, whereas models for morphogenetic movements are often force-based, involving mechanics and interacting cell behaviors [129] (so-called cell regulatory networks [136]). While we summarized results deconstructing these different dynamical systems independently, molecular and mechanical mechanisms interact across scales to propel trajectories in both spaces simultaneously (Fig. 2C). This raises new questions: How are canalization in pattern and form intertwined? Can coupling between morphogenesis and fate patterning elucidate synergies and developmental constraints? When morphogenesis and differentiation are inherently coupled, answering these questions requires integrated theoretical frameworks to predict perturbations to both aspects and elucidate relevant feedback. Here, we review literature on integrated morphogenesis and cell differentiation and suggest avenues towards unification.

Some feedback of $g_{i}(t)$ on $x_{i}(t)$ is relatively cell-autonomous, as in the individual migration of differentiating cells [137]. Instead, collective feedback between $\left\{x_{i}(t)\right\}$ and $\left\{g_{i}(t)\right\}$ involve spatial molecular or mechanical interactions among multiple cells [138]. For example, cell-adhesion patterns downstream of $\left\{g_{i}(t)\right\}$ control cell sorting [139,140] and collective morphogenetic movements [141,142]. Conversely, tissue density may influence morphogen diffusivity [143], patterning precision [144], and lateral inhibition-based patterning [145]. Broadly, morphogenesis may influence fates via mechanics—i.e., mechanotransduction (reviewed in [146,147])—and through its kinematic effects. The latter affect distance-dependent cell-cell interaction mechanisms [148,149]. Below, we focus on how dynamic tissues may affect diffusing morphogens controlling fate decisions.

### 6.1. Dynamic tissue patterning

Gene expression and positional trajectories are intimately related because the signals cells receive from other cells depend on cell positions. Experimental evidence points to fate decisions depending on morphogen exposure over time $m_{i}(t)$ [150-155], consistent with their role as control parameters. As [15] notes, understanding fate coordination *in-vivo* requires accounting for spatiotemporal morphogen dynamics $\left\{m_{i}(t)\right\}$. Spatial coordinates can act as effective control parameters for decision space dynamics [60], but only if positions reliably reflect signaling environments. Positional information provides a framework for justifying this correspondence in static tissues, but cells in dynamic tissues need to integrate this dynamic signaling information along $x_{i}(t)$ [156]. This creates opportunities for morphogenesis to play a generative role in patterning [152,157-161]. Morphogenetic movements can reshape or rearrange morphogen patterns [162] and affect fate decisions by influencing the dynamic morphogen exposure of moving cells [152,157]. Below, we review how cell trajectories $\left\{x_{i}(t)\right\}$ and the DM shape morphogen patterns and exposure $\left\{m_{i}(t)\right\}$, upstream of fate decisions.

Early models of coupled morphogenesis and patterning include the progress zone model in vertebrate limb development, where fate decisions depend on the time spent in a signaling region before exiting [163,164]. Similarly, in the time-space translation hypothesis for vertebrate anterior-posterior patterning, initial Hox gene activation depends on timed entry—via convergent extension—of an inductive zone [165,166], explaining the spatial and temporal collinearity of Hox gene expression patterns [167]. The exact mechanisms and spatiotemporal dynamics of this inductive zone remain unclear, and secreted factors have been implicated in both Hox expression activation [168] and progression [169], suggesting a dynamic interplay between positioning, morphogen patterning, and gene regulation.

In contrast with timed entries and exits of static signaling environments, morphogen patterns can be dynamic and depend on morphogenetic movements [170]. Growth is the most common morphogenetic motif integrated into patterning models [171]. In digit formation, growth controls the size and number of pattern elements [172-174], reducing variability in line with theoretical models [175]. Across morphogen gradient models, growth also facilitates pattern scaling [176-179] (reviewed in [180]). Recent approaches attempt to deconfound the effects of complex cell motion on patterning by integrating live-imaged cell trajectories $\left\{x_{i}(t)\right\}$ with stained embryo snapshots of $\left\{m_{i}\right\}$ and $\left\{g_{i}\right\}$ at different times. This enabled approximating $\left\{m_{i}(t)\right\}$ and $\left\{g_{i}(t)\right\}$ and inferring gene regulatory dynamics, predicting various experimental perturbations in zebrafish [152,181]. Similar approaches enabled approximating $\left\{g_{i}(t)\right\}$ in the deforming mouse limb bud and neural tube [182]. The dependence of fate decisions on dynamic signaling requires accounting for $m_{i}(t)$ along cell trajectories instead of m(x) at fixed positions (Fig. 4B), which are the default coordinates in reaction-diffusion models, even when advection is present. Theoretically, this calls for patterning frameworks in Lagrangian (instead of Eulerian) spatial coordinates (Fig. 4A).

### 6.2. Patterning equations in the cell frame

A recent work reformulating standard Eulerian advection-reaction-diffusion patterning equations in Lagrangian coordinates (the cell frame) elucidates how tissue deformation mediates morphogen patterning [112]. In patterning, secreted morphogens diffuse across cells, but morphogenesis changes their proximity, resulting in dynamic cell-cell interaction ranges. These effects appear directly when patterning equations are expressed in the cell frame, with tissue deformation accelerating, decelerating and providing anisotropy (directional dependence) to interaction ranges. For example, convergent extension progressively enhances diffusive fluxes between converging cells and reduces it between those separating [112]. This framework, using readily available experimental data on tissue flows, *i)* de-confounds the effects of motion by describing morphogen exposure histories—inputs to fate decisions—in cells’ moving frames; *ii)* rationalizes the effect of general tissue deformation on dynamic cell-cell interaction ranges; *iii)* provides nondimensional parameters to identify when and where deformations might affect morphogen patterning.

Repellers and attractors in the DM define regions of maximum deformation, specifically spatial separation and convergence. Theoretically, this implies that they also identify the strongest barriers and enhancers of morphogen diffusive fluxes in the cell frame, thereby reshaping cell-cell interactions within [$t_{0}$, $t_{0}+T$] (Fig. 4C) [112]. This finding reveals a relationship between morphogenetic flows and morphogen patterning. Quantification of the DM can therefore be used to assess if, when, and where morphogenesis may affect patterning. It also suggests possible connections between positional attractors and repellers—shaping morphogen patterns—and their counterparts in decision space (Fig. 4D). Repellers—reducing interaction ranges—may support diverging cell fates, consistent with recent experimental evidence in chick [117] and zebrafish [12] embryos, while attractors—enhancing interaction ranges—support cell fate induction (Fig. 4E). The specific interplay between morphogenetic movements and fate decisions depends on the rate, timing, and duration of tissue deformation, local morphogen production, transport, and removal mechanisms [50], signal transduction, and fate transitions. Still, the morphogenetic constraints on intercellular communication are general. For example, tissue expansion gradually reduces communication between initially nearby cells [183], regardless of their specific signaling mechanisms. The geometric structure of decision spaces can rationalize nonlinear mappings between morphogen exposure and discrete cell fates ($m_{i}\to g_{i}$). Dynamic morphoskeletons, directly implicated in morphogen patterning equations in cells’ moving frames [112], similarly help rationalize the complex mapping between morphogenetic movements and morphogen exposure histories ($\left\{x_{i}(t)\}\to \{m_{i}(t)\right\}$). Characterizing in detail if and how geometric structures in position and decision spaces interact (e.g., 4E) requires experimental and theoretical advances. While challenging to measure extracellular morphogen dynamics directly—even in static tissues—techniques are emerging to control synthetic morphogen gradients [184-186] (reviewed in [187]). Mathematical modeling is also crucial to understanding mechanochemical feedback through which morphogen patterns stabilize [188] or initiate [189] robust morphogenetic movements. Discrete cell-scale modeling may also prove essential for representing certain patterning mechanisms, such as Notch-Delta lateral inhibition [130,145]. Ultimately, unifying spatial and gene expression trajectories is crucial to understand how the right cell types end up in the right places. Historically, fate maps have provided a general tool to track where initial configurations of cells go—comparable to the flow map—and what fates they acquire (reviewed in [190]). Consistent fate maps reflect the deterministic component of morphogenetic movements, while inconsistencies arise from random cell mixing and imprecise movements [191]. Non-random movement is needed for morphogenesis, but noisy cell motion can help homogenize signaling environments [61] or sort out spatial patterns [139,192]. Specification maps complement fate maps by revealing the fates cells would acquire if initially explanted and deprived of the signals received along their ordinary trajectories. Discrepancies between fate and specification maps suggest that external signaling influences fate decisions [1,193]—i.e., molecular or mechanical signals $m_{i}(t)$—but do not clarify what signals were relevant or which cells interacted within the competence window. Studying flow maps’ properties may help rationalize these differences by connecting patterns of convergence and separation in space (the DM) to diverging fate domains (Fig. 4F) via morphogenesis-mediated cell-cell communication [112].

## 7. Developmental constraints

Dynamical systems theory provides a general framework and vocabulary to study complex dynamic processes such as embryonic development. Understanding development requires decomposing its complexity into distinct sub-processes with simpler structured dynamics and elucidating how they connect and constrain each other. From a dynamical systems perspective, constraints can be categorized as internal (phase space geometry: manifolds organizing trajectories in phase space) or external (boundary conditions, control parameters, or initial conditions), and this designation depends on how development is partitioned. For the whole embryo, all constraints are internal except for maternal and environmental factors, helping to explain biases in phenotypic variation [194]. A differentiating cell exhibits internal regulatory constraints, subject to external signaling constraints and initial conditions, both to remain functional and avoid the aberrant attracting states associated with cancer [195,196]. At tissue scales, external constraints can be architectural, including geometric and mechanical boundary conditions that drive internal morphogenesis [138,197], while initial conditions are the constraints supplied by prior stages [105].

How one partitions development in space and time into distinct dynamical systems should account for the extent and timing of internal self-organization (e.g. among positions and decisions), and the experimental manipulability of external constraints. This is especially challenging for integrated models of morphogenesis and differentiation, because the processes may be concurrent, and strong internal feedback limits external control over many mechanical and molecular parameters. Addressing this requires measurements and model-experiment perturbations at appropriate levels of organization [198], inviting both new technologies [199], time-dependent molecular and mechanical perturbations, and classic experimental embryology techniques like tissue grafting, removal, or embedding [200]. Alberch pioneered the idea that abnormal developmental trajectories can reveal developmental constraints. Complementary to perturbing normal development, embryo explants and stem-cell based embryo models systematically relax external constraints, exhibiting reduced developmental potential, but retaining some self-organizing capacities [157,201-204]. Relaxing and reintroducing *in-vivo* external constraints can help elucidate their interplay with the internal constraints of development [104,159]. The existence of developmental constraints indicates that development unfolds in far fewer dimensions than the data we collect. As data’s dimensionality grows, geometric reasoning and integrated theoretical frameworks provide a powerful way to organize complicated dynamical data into an interpretable schematic of development’s stable plan.
